# Micro-patterning of spintronic emitters enables ultrabroadband structured terahertz radiation

**DOI:** 10.1038/s41377-024-01579-y

**Published:** 2024-09-05

**Authors:** Hou-Tong Chen

**Affiliations:** Center for Integrated Nanotechnologies, Los Alamos National Laboratory, Los Alamos, NM 87545 USA

**Keywords:** Metamaterials, Terahertz optics

## Abstract

Structured light beams offer promising properties for a variety of applications, but the generation of broadband structured light remains a challenge. New opportunities are emerging in the terahertz frequency range owing to recent progress in light-driven ultrafast vectorial currents through spatially patterning spintronic and optoelectronic systems.

Structured light refers to optical beams with spatially variant intensity, polarization, and/or phase in the beam cross-section. Radially and azimuthally polarized beams are examples of a general class of cylindrical vector beams with axial symmetry in both amplitude and phase^1^. Their unique and promising properties can find important applications, such as a longitudinal field component^2^ that is stronger and more localized^3,4^, thus enabling tighter focusing for microscopy imaging^5^, particle acceleration^6,7^, and tailored light–matter interactions^8^. A variety of approaches have been developed in the optical region to convert a homogeneous Gaussian beam to a structured beam, and many of them have been adapted to the terahertz (THz) frequency range^9^, but they typically operate within a limited spectral band. The generation of truly achromatic and arbitrary structured beams remains a great challenge in the THz frequency range, where the ultrabroad spectrum in ultrafast THz radiation represents the most important and advantageous characteristic. In the recent issue of *eLight*, Wang et al.^10^ address this challenge by developing a versatile method that directly generates some complex structured THz beams by using programmable spintronic emitters.

The research work is built on ultrafast spintronic THz emitters that have been developed during the last decade. The simplest spintronic THz emitters^11,12^ are thin film heterostructures consisting of a ferromagnetic (FM) layer with in-plane magnetization and a nonmagnetic (NM) metal layer with strong spin–orbital coupling. Femtosecond laser excitation results in transient demagnetization in the FM film, and also induces a net spin transport from the FM to the NM metal layer due to a gradient of the electronic temperature and a spin voltage between the FM and NM layers. Transverse charge/spin currents are then generated in the NM metal owing to the inverse spin Hall effect, which radiate ultrafast THz pulses to free space. The direction of the charge current is determined by and perpendicular to both the spin current along surface normal and the in-plane magnetization of the FM layer. To be used as THz emitters, an external magnetic field is usually applied to induce a uniform magnetization, which may otherwise form domains with random magnetization directions. Consequently, the emitted THz radiation typically possesses a spatially homogeneous linear polarization state although the polarization angle is tunable by rotating the orientation of the spintronic THz emitter.

Generating structured THz beams requires spatially variant current directions with subwavelength resolutions (i.e., a few tens of micrometers or less). Wang et al. accomplish this through patterning magnetization directions by innovatively employing a laser-assisted magnetic programming (LAMP) method^13^. To enable LAMP, an additional layer of 6-nm-thick IrMn antiferromagnetic (AFM) film is added beneath the heterostructure consisting of 3-nm-thick Fe_21_Ni_79_ as the FM layer and 2-nm-thick Pt heavy metal as the NM layer (see Fig. 1). By applying an external magnetic field and owing to the exchange coupling with the FM layer, the AFM spin direction can be locally rearranged during the laser writing, and consequently pin the local FM magnetization direction after removing the external magnetic field. In such a way, the authors create spatial patterns of magnetization to generate a variety of structured THz fields, including a linear gradient of Pancharatnam–Berry phase for separating the left- and right-handed circular polarizations, a radial magnetization for azimuthal THz vector beam (see Fig. 1), and a more complex spatial pattern for generating a THz beam containing all the states of polarization in the beam cross-section.

**Fig. 1:**
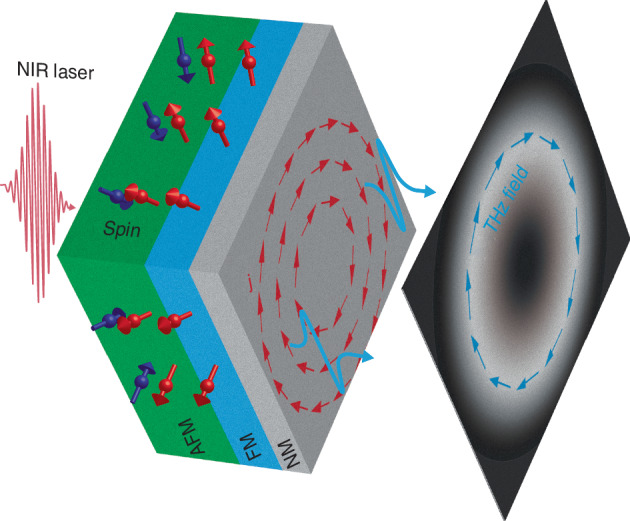
A micro-patterned spintronic emitter for generating complex structured THz fields. The antiferromagnetic (AFM), ferromagnetic (FM), and nonmagnetic (NM) metallic heterostructure allows spatial patterning of magnetization (in the FM layer) and charge current (in the NM layer) directions with microscale resolutions, leading to a new capability of generating complex structured THz fields, e.g., azimuthally polarized ultrabroadband THz vector beams

The work by Wang et al. is impressive in a few perspectives. The demonstrated spintronic THz emitters are programmable to accomplish arbitrary structured THz fields, some of which may be difficult to realize through conventional polarization conversion approaches. For vector beams, all frequency components of the generated THz fields are included, thus they are ultrabroadband, in contrast to those through conversion with a limited spectral bandwidth. This also makes them an excellent platform for the observation of toroidal pulses^14^. Compared with recent work on generating THz vector beams using graphene-based nano-optoelectronic metasurfaces^15^, this work offers a significant advantage in manufacturing large-area devices (centimeter or more), which may allow the generation of high-intensity structured THz beams.

Given the demonstrated potential of micro-/nano-scale spatial patterning for manipulating local THz linear polarization states^10,15^, it is highly plausible that advancements in this field will pave the way for the generation of a diverse array of novel polarization states and structured THz beams. For instance, the realization of ultrabroadband circular polarization states and the corresponding vortex beams endowed with pure orbital angular momentum, may be on the horizon. These innovations hold the promise of unlocking a plethora of intriguing applications across various scientific and technological domains. While the path forward necessitates innovative and creative thinking, the groundwork laid by these pioneering studies provides a robust and inspiring foundation upon which future explorations can be built.
